# Family Functioning in Families Affected by Parental Mental Illness: Parent, Child, and Clinician Ratings

**DOI:** 10.3390/ijerph18157985

**Published:** 2021-07-28

**Authors:** Marlit Sell, Anne Daubmann, Holger Zapf, Bonnie Adema, Mareike Busmann, Maja Stiawa, Sibylle M. Winter, Martin Lambert, Karl Wegscheider, Silke Wiegand-Grefe

**Affiliations:** 1 Department of Child and Adolescent Psychiatry and Psychotherapy, University Medical Center Hamburg-Eppendorf, 20251 Hamburg, Germany; h.zapf@uke.de (H.Z.); b.filter@uke.de (B.A.); m.busmann@uke.de (M.B.); 2 Department of Medical Biometry and Epidemiology, University Medical Center Hamburg-Eppendorf, 20251 Hamburg, Germany; a.daubmann@uke.de (A.D.); k.wegscheider@uke.de (K.W.); 3 Department of Psychiatry and Psychotherapy II, Ulm University, 89312 Günzburg, Germany; maja.stiawa@uni-ulm.de; 4 Department of Child and Adolescent Psychiatry, Psychosomatics and Psychotherapy, Charité—Universitätsmedizin Berlin, Campus Virchow-Klinikum, 13353 Berlin, Germany; sibylle.winter@charite.de; 5 Department of Psychiatry and Psychotherapy, University Medical Center Hamburg-Eppendorf, 20251 Hamburg, Germany; lambert@uke.de

**Keywords:** parental mental illness, family functioning, multiple informants, informant discrepancies

## Abstract

Family functioning is often impaired in families with a parent with mental illness and is linked to child mental health. This study aims to gain a better understanding of family functioning in affected families by comparing ratings among family members and by analyzing associations with clinician-rated family functioning. The cross-sectional sample comprised 210 families with ratings of 207 patients, 139 partners, and 100 children. Parents with a mental illness as well as their partners and children completed the German version of the Family Assessment Measure (FAM). Clinician ratings were obtained by the Global Assessment of Relational Functioning Scale (GARF). We conducted several mixed models to compare ratings of family functioning while accounting for family cluster. Family dysfunction was consistently elevated compared to a normative sample. On several domains, parents with a mental illness perceived family functioning to be worse compared to their partners and children. Partners and children did not differ in their perceptions of family functioning. Ratings of family members were moderately associated with clinician ratings. We discuss the importance of multi-informant assessment of family functioning and the implementation of family-based interventions for families with a parent with mental illness.

## 1. Introduction

It is estimated that around 12 to 45% of adults attending psychiatric services are parents to minor children [1,2]. Parental mental illness not only affects the individual but has consequences for their children, partners, and the family environment [3]. In previous research, parental mental illness has been connected to marital distress and poor family relationships [4]. Children whose parents have a mental illness are at high risk for the development of psychological symptoms like depression and anxiety as well as poor social functioning [5,6]. The results of long-term studies also reveal that this at-risk status continues throughout the lifespan [7,8]. Besides genetics, environmental factors like parent–child interaction, social support, and family functioning are involved in the intergenerational transmission of mental health problems [1,9,10,11].

Family functioning is a multi-faceted construct referring to the dynamics within the family [12]. Whereas some studies focus on narrower concepts related to family functioning, such as parenting behaviors, the term is usually used to describe the collective functioning of a family unit [13]. There are different conceptual models of family functioning. One important theoretical framework is the Process Model of Family Functioning [14,15]. It was derived from systems theory and describes the way families accomplish important tasks to satisfy the needs of their members. According to this model, aspects of family functioning include, for example, communication, affective expression, and role performance [12].

Impaired family functioning has been found for various parental mental illnesses such as major depression and bipolar disorders [13,16,17,18], as well as psychotic [19] and anxiety disorders [20]. Moreover, personality disorders are characterized by disturbances in close relationships and have accordingly been linked to poor family functioning. For example, Miller et al. [21] found that in a sample of depressed patients, the level of personality disorder symptomatology was strongly associated with family dysfunction. Furthermore, indicators of patients’ illness severity are related to family functioning. For instance, in families with bipolar patients, years of illness, number of suicide attempts, and severity of manic symptoms are related to negative family environment [16]. Across several parental mental illnesses, overall symptom severity is linked to higher levels of family dysfunction [22,23,24].

Collecting information from multiple informants is considered important when examining child and family variables since it provides a fuller picture and more valid assessment [25,26,27]. However, using only parent or child reports of family functioning is still a widely used practice in empirical studies [22,28]. When several family members are asked to rate characteristics of their family, their ratings often diverge [29,30]. Discrepancies in family constructs are not merely a product of measurement error, but instead may reflect an individual’s own perspective of the family and convey meaningful information [28,29].

Studies in the general population typically report low to moderate correspondence among parents’ and adolescents’ ratings of family functioning [30]. Several studies found children’s and adolescents’ ratings of family functioning to be more negative than their parents’ ratings [3,31,32,33]. For example, in a study by Ohannessian, Lerner, Lerner, and von Eye [31], adolescents reported significantly lower levels of family cohesion and family adjustment than their parents. Such differences might be explained by normative changes in autonomy development in adolescence [33,34].

Compared to findings from non-clinical samples, data on cross-informant agreement for samples selected for psychopathology are limited [35]. Findings indicate that discrepancies in ratings of family functioning are especially pronounced when families are facing stressful circumstances like parental illness [32]. The presence of mental health problems has been found to be a relevant predictor of agreement in family constructs like parenting behavior [36] and also overall family functioning [3].

Studies have shown that parents with a mental illness may have biased perceptions about their children and family. According to the depression-distortion hypothesis, depressive symptoms of an informant may negatively influence their rating of their child [37,38]. Such a bias has mainly been investigated for parental depressive symptoms but has also been found for overall psychopathology leading to a more generalized model of psychopathology distortion [39]. This model assumes that parents with a mental illness hold negative schemas which may also affect how they view their family [39,40]. A negatively biased perception in parental depression has also been found for ratings of family functioning. For example, Pérez, Coo, and Irarrázaval [3] compared mothers’ and adolescents’ ratings of family functioning. They found that, in general, adolescents had a less favorable view of family functioning than their mothers. However, as mothers’ depressive symptoms increased, adolescents viewed family cohesion in a more favorable way than their mothers. The authors conclude that maternal depression may have negatively biased the mothers’ perceptions of family functioning, leading to this pattern of disagreement.

Concerning ratings of patients with a mental illness and their partners, researchers report moderate levels of agreement on most family functioning dimensions for patients with alcohol-abuse [41] as well as depression [23]. Regarding differences between ratings, findings are mixed. Osterman and Grubic [42] found no differences between patient and partner rated family functioning in a sample of alcohol-addicted patients in recovery. However, a study by Wang and Zhao [23] indicates that depressed patients report significantly greater impairments on the family functioning dimension of problem-solving.

While previous studies compared patients’ ratings of family functioning with those of either their partner [23,41,42] or their children [3,13], few studies so far have simultaneously investigated several family members’ ratings, including those of both partners and children. The studies that included several family members’ ratings did not directly compare ratings of partners and children [35,43]. Further limitations raised by previous studies on informant discrepancies regard the focus on discrepancies between mothers’ and children’s perspectives and a lack of studies that also take fathers’ perceptions into account [3,13,33,34]. Also, researchers have recommended incorporating external ratings of family functioning since self-report questionnaires may be influenced by social desirability bias [13,16,33]. An external assessment of family functioning by clinicians can be regarded a useful reference point since it may differentiate between actual dysfunction and potential biases of family members [44].

Advancing knowledge on differences between family members’ perceptions of family functioning can provide information relevant for assessment and treatment purposes [35]. This study aims to further understand family functioning in families affected by parental mental illness by comparing different members’ perceptions of family functioning.

The following research questions were addressed: (1) Do family members in families affected by parental mental illness report impaired family functioning compared to normative data? (2) Do mentally ill parents, partners, and children differ in their perceptions of family functioning? (3) How are family members’ ratings of family functioning associated with external ratings of clinicians?

We expected that family functioning would be impaired across different parental mental illnesses. Moreover, we expected that there would be significant discrepancies between the ratings of mentally ill parents, their partners, and their children on family functioning, with mentally ill parents indicating higher levels of family dysfunction than their partners and children. We expected family members’ ratings of family functioning to be associated with clinicians’ ratings. In addition, we exploratively tested for possible moderator effects of family members’ gender, parental illness severity, and social desirability.

## 2. Materials and Methods

### 2.1. Study Design

This study utilized data from the baseline assessment of a randomized controlled trial (“Implementation and evaluation of a family-based intervention program for children of mentally ill parents: a randomized controlled multicenter trial”) funded by the German Federal Ministry of Education and Research (BMBF). Details of the study have been reported elsewhere [45]. Data were gathered between 2014 and 2017. Study sites were the following seven clinical centers based in Germany and Switzerland: Hamburg (University Medical Center Hamburg-Eppendorf, Germany), Leipzig (University Medical Center Leipzig, Germany), Ulm-Günzburg (Ulm University, Department of Psychiatry and Psychotherapy II, Germany), Wiesbaden-Rheingau (Medical Center Vitos Clinic, Germany), Gütersloh-Paderborn (LWL Community Hospitals, Germany), Berlin (Charité—Universitätsmedizin Berlin, Germany) and Winterthur (Center of Social Pediatrics, Switzerland). Among other study variables, family functioning was assessed by parents with a mental illness, their partners, and children, as well as clinicians with standardized psychometric instruments. The study was approved by the Ethics Committee of the Chamber of Physicians in Hamburg, Germany (PV4744).

### 2.2. Sample

Families were included if one parent in the family (the “patient”) met the diagnostic criteria of a mental disorder according to ICD-10. Parental diagnoses were rated by an attending clinician. Further, written informed consent to participate in the study and sufficient knowledge of the German language by parents and children were required. Acute severe parental psychiatric symptoms with indication for inpatient treatment were an exclusion criterion.

The overall trial sample consisted of *N* = 216 families including 216 patients, 145 partners and 338 children. Self-reports were obtained for 143 children, of which 37 were not considered for the current analyses because the children were younger than 12 years, which was outside the age range of the normative sample of the administered questionnaire. Further, the data of 9 patients, 6 partners and 6 children were excluded due to complete or extensive (>30% of items) missing data for the baseline assessment. The resulting sample comprised 210 families, including ratings of 207 patients, 139 partners and 100 children. Among the families, 11% provided ratings of more than one child per family. The mean age of patients was *M* = 39.99 years (*SD* = 7.15, range: 23–57), of partners *M* = 40.29 years (*SD* = 6.93, range: 23–59), and of children *M* = 14.87 years (*SD* = 2.03, range: 12–19). The most frequent parental mental illnesses in our sample were affective disorders (ICD-10, F30–F39), followed by personality disorders (ICD-10, F60–F69), and neurotic, stress-related, and somatoform disorders (ICD-10; F40–F48). Further demographic and clinical characteristics of the sample are displayed in Table 1.

The sample of clinicians was comprised of *N* = 31 (79.2% female) staff members of the seven participating clinical centers. All clinical raters had at least master’s-level psychology training and received specific training for study purposes. Clinicians were supervised by advanced or senior psychotherapists or psychologists.

### 2.3. Measures

#### 2.3.1. Family Members’ Perspectives

Patients, partners, and children rated family functioning on the German version of the Family Assessment Measure (FAM) [46], a questionnaire based on the ‘Process Model of Family Functioning’ [14,15]. The questionnaire comprises 28 items and ratings are done on a 4-point scale ranging from 0 (“completely true”) to 3 (“not true at all”). The questionnaire consists of the following seven subscales: Task Accomplishment, Role Performance, Communication, Emotionality, Affective Involvement, Control, Values and Norms. A total sum score can be calculated for the analyses reflecting the global functioning of the family. On each score, higher values represent greater family dysfunction. In addition, the FAM contains the control scale Social Desirability with six items, which are answered on a 4-point scale.

The FAM has been normed in a sample of healthy families and raw scores can be transformed to T-values (*M* = 50, *SD* = 10). T-scores greater than 60 represent difficulties in family functioning. Normative data of the FAM are based on 218 German families with ratings of 413 parents and 75 children. Families could be included in the reference sample if they had not received psychiatric or psychotherapeutic treatment in the last 5 years. Comparisons with normative data can be obtained for different life-cycle stages of the family including families with younger children (oldest child in the household between 1 and 11 years) and older children (oldest child in the household >12 years) [46,47]. The FAM has good concurrent and convergent validity and very good content validity [47]. Estimates of internal consistency for the different subscales in this sample were the following: Global Family Functioning, *α* = 0.93; Task Accomplishment, *α* = 0.72; Role Performance, *α* = 0.71; Communication, *α* = 0.68; Emotionality, *α* = 0.61; Affective Involvement, *α* = 0.71; Control, *α* = 0.64; Values and Norms, *α* = 0.66; Social Desirability, *α* = 0.78.

#### 2.3.2. Clinician Perspective

Family functioning from the clinicians’ perspective was assessed with the “Global Assessment of Relational Functioning Scale” (GARF) [48]. The scale comprises a composite measure of the three family functioning dimensions: joint problem solving, organization, and emotional climate. Levels of functioning are rated on a 1–100-point scale. Anchor-point descriptions are available at five major levels of functioning. The lowest range of values between 1 and 20 indicates that the “relational unit has become too dysfunctional to retain continuity of contact and attachment”, whereas the highest category of functioning with values between 81 and 100 reflects that the “relational unit is functioning satisfactorily”. Assessments were completed on the basis of interviews with the families. Regarding the scales’ psychometric properties, studies in clinical samples reveal high interrater reliability [48,49] and good concurrent and convergent validity [49,50].

Clinicians also rated patients’ illness severity based on the Clinical Global Impression Scale (CGI) [51]. The single-item measure assesses symptom severity on a seven-point scale ranging from 1 (“Not at all ill”) to 7 (“Among the most extremely ill patients”). The CGI is widely used in clinical research and practice and has shown good validity in several clinical populations [52,53].

### 2.4. Statistical Analyses

Due to the hierarchical structure (subjects clustered within families), we used mixed models to analyze the data [54]. To answer our first research question, we converted raw scores to T-scores according to the norm tables of the FAM and compared family members’ ratings to the distribution of the normative sample with a reference value of T = 50. To answer our second research question of whether family members differ in their perception of family functioning, we analyzed pairwise comparisons between family members’ ratings of family functioning based on estimated marginal means. Finally, for our last research question, we modeled the association between family members’ ratings (FAM) and clinician’s ratings (GARF) of family functioning. The restricted maximum likelihood estimation (REML) was used for all analyses. Missing values were imputed according to the Expectation–Maximization algorithm (EM) [55]. Analyses were performed using IBM SPSS statistics, version 26.

## 3. Results

### 3.1. Descriptive Analyses and Comparison with Normative Data

Preliminary tests of skewness (range from −0.08 to 0.52) and kurtosis (range from −0.53 to 0.15) for dependent variables suggested that non-normality of the data was not a concern with all values being in the range of −1 and +1; [56]. Means and standard deviations for the several scales of the FAM from the family members’ perspectives are displayed in Table 2. Family members’ mean score on the FAM control scale, Social Desirability, was 6.43 (*SD* = 3.45). Clinicians’ ratings on the GARF had a mean of 65.13 (*SD* = 17.47) and ratings on the CGI had a mean of 4.19 (*SD* = 0.89).

Results on our first research question regarding family functioning compared to normative data are displayed in Table 3. For all scales, family members differed from the reference population (reference value = 50) with impaired family functioning compared to the norm. For most scales, the adjusted mean T-value was above 60 representing difficulties in the family. The adjusted mean T-value for Social Desirability was 41.88 (95% CI = 40.74 to 43.01) indicating lower levels of social desirability than the reference population (*p* < 0.001).

### 3.2. Differences between Family Members’ Ratings

Results regarding our second research question on differences between family members’ ratings are displayed in Table 4. There was a significant difference between patient and partner ratings in all scales of family functioning. Patients reported higher levels of family dysfunction than their partners. For global family functioning and most subscales, patient and child ratings of family functioning also differed, with patients reporting higher family dysfunction than their children. Only for two subscales, Emotionality as well as Values and Norms, was there no significant difference between ratings of patients and children. Ratings of partners and children did not significantly differ for any of the family functioning scales.

Patients’ illness severity (CGI) and family members’ gender were included in subsequent moderator analyses. There were no significant interaction effects between family member and patients’ illness severity on FAM scores (Global Family Functioning: *F*(2, 255.59) = 0.66, *p* = 0.516; Task Accomplishment: *F*(2, 266.27) = 1.15, *p* = 0.319; Role Performance: *F*(2, 266.08) = 0.72, *p* = 0.488; Communication: *F*(2, 271.24) = 1.45, *p* = 0.236; Emotionality: *F*(2, 270.08) = 1.57, *p* = 0.210; Affective Involvement: *F*(2, 257.32) = 0.01, *p* = 0.993; Control: *F*(2, 265.88) = 1.57, *p* = 0.210; Values and Norms: *F*(2, 262.76) = 0.19, *p* = 0.829).

Furthermore, interaction terms between gender and family member turned out to be non-significant (Global Family Functioning: *F*(2, 317.80) = 0.50, *p* = 0.609; Task Accomplishment: *F*(2, 321.99) = 0.25, *p* = 0.779; Role Performance: *F*(2, 316.04) = 0.07, *p* = 0.928; Communication: *F*(2, 316.50) = 1.77, *p* = 0.172; Emotionality: *F*(2, 319.76) = 0.95, *p* = 0.388; Affective Involvement: *F*(2, 316.46) = 0.98, *p* = 0.377; Control: *F*(2, 312.50) = 0.94, *p* = 0.390; Values and Norms: *F*(2, 313.67) = 0.35, *p* = 0.702).

### 3.3. Association between Family Members’ and Clinican Ratings

Bivariate Pearson correlations between family members’ ratings of global family functioning on the FAM and clinicians’ ratings on the GARF were the following: patient-clinician, *r* = −0.33, *p* < 0.001; partner-clinician, *r* = −0.34, *p* < 0.001; child-clinician, *r* = −0.25, *p* = 0.029.

To account for family cluster in our analyses, we conducted a mixed model entering the GARF score as regressor and the FAM scores as regressand while adjusting for the type of family member. Clinician ratings on the GARF were significantly related to FAM ratings (*b* = −0.26, *p* < 0.001). On average, one unit increase in clinician-rated GARF was associated with a decrease by 0.26 units in family members’ ratings on the FAM. Thus, higher family functioning rated by the clinician (indicated by higher levels on the GARF) was also related to higher family functioning rated by family members (indicated by lower levels on the FAM).

To assess whether associations between GARF and FAM differed depending on the family member (patient, partner, child), we entered an interaction term between family member and GARF-score in the model. The interaction term turned out to be non-significant (*F*(2, 264.55) = 0.94, *p* = 0.393). We further tested whether the association between GARF and FAM differed depending on the level of family members’ social desirability by entering in an interaction term between the Social Desirability score and the GARF-score. The interaction term was not significant (*F*(1, 342.41) < 0.01, *p* = 0.989).

## 4. Discussion

In our study, we aimed to assess family functioning in families with a parent with mental illness and to compare ratings of family members among each other, to normative data, and to external ratings of clinicians. As expected, families displayed impaired family functioning compared to a normative sample. This finding is in line with a series of studies showing that family functioning is reduced when a family member has a mental illness [13,16,17,18]. Thus, interventions not only addressing the patient, but the family as a whole, are essential. Family-based interventions for families affected by parental mental illness have shown long-term improvements in levels of family functioning. For example, Beardslee et al. [57] report sustained improvements in family functioning for two family-based preventive interventions in their 5-year follow-up.

When comparing ratings of family members, parents with a mental illness consistently reported more severe family dysfunction than their partners. This result is in line with the finding of Wang and Zhao [23] that depressed patients in their sample reported more severe impairments of family functioning than their partners. While Wang and Zhao [23] report this difference only for the subscale of problem-solving, we found a difference for all scales of family functioning in our study. One possible reason for this difference regards the scope of psychopathology since we included severe parental mental illnesses and comorbidities other than depression. Mentally ill parents also reported higher impairment on most dimensions of family functioning than their children. Both results are in line with our expectations and may be indicative of a negatively-biased view of patients with a mental illness, as has been suggested in previous research regarding child [37,38] as well as family outcomes [3]. However, there is no “gold standard” for the correct rating among family members and the common approach is to view each informant as a valuable source of information [39]. Hence, alternative interpretations for the observed differences must be considered. For example, partners and children may under-report family dysfunction as well since family members may tend to conceal family problems to protect the parent with mental illness [58].

Partners and children of patients did not differ on any dimension of family functioning, indicating that both types of family members report similar levels of family functioning. To the best of our knowledge, previous studies in samples of families affected by parental mental illness did not directly compare ratings of partners and children. Due to this, we cannot collate our findings with those from previous studies. A replication of this specific comparison in future studies is necessary to verify our result.

Our analysis yielded low to moderate correlations between family functioning ratings of family members and clinicians. Ratings of family members and clinicians were also related when controlling for family cluster. The magnitude of correlations is in line with previous research [59,60]. In a sample of patients in family therapy, Denton, Nakonezny, and Burwell [60] likewise reported that ratings of family members and clinicians were significantly associated when adjusting for family cluster in mixed model analyses. In our subsequent analyses, we found that the association between ratings of family members and clinicians did not differ depending on the specific family member. This is in line with the findings of Sheets and Miller [59], who also found similar correlations for ratings of patients and partners with external ratings but in contrast to findings of Wang and Zhao [23]. The latter reported greater agreement between patients and clinicians than between their partners and clinicians. We further included family members’ level of social desirability in our subsequent analyses. One reason researchers have recommended using external ratings of family functioning is to correct for a possible social desirability bias in self-reports [13,16,33]. However, our results suggest that the link between family members’ and clinicians’ ratings does not differ depending on the social desirability displayed by family members. Our comparison to normative data further indicates that participants in our study displayed relatively low levels of social desirability. Since social desirability can be regarded as a multifaceted construct [61], considering different aspects of this construct may lead to more insights in future investigations.

There are several limitations to our study. First, we used normative data of the FAM to compare family functioning of our sample to the general population. While using normative data to compare clinical to non-clinical samples is a commonly used approach [18,62,63], future studies should also include the simultaneous assessment of a healthy control group. This is especially relevant since using a control group enables one to account for other possible confounding variables [62]. Also, the way families are constituted and function changes considerably over time [64] and normative data become less representative. Second, we analyzed a mixed sample of families with different parental diagnoses with the majority of patients having an affective disorder. Our findings may therefore be more representative for families affected by parental affective disorders. Future studies would benefit from larger samples with a more balanced distribution of parental mental illnesses and standardized diagnostic assessments for all family members. Such studies might also differentiate between parental diagnosis groups. There is some evidence for differences in the agreement between family members’ ratings of family functioning depending on the specific mental illness. For example, a study by Weinstock, Wenze, Munroe, and Miller [35] showed a lower degree of concordance in family members’ reports of family functioning in a sample of patients with bipolar disorders than in a sample of patients with major depressive disorders. Third, participants in this study were recruited for participation in a family-based intervention for families affected by parental mental illness. The assessment of family functioning was done before the start of the study interventions. However, the family members in our sample may differ from those not seeking help in family-based interventions.

Among the strengths of our study is that besides family members’ ratings, we also took into account clinician ratings, as recommended by several researchers [13,16,33]. We considered both maternal as well as paternal ratings, whereas past research mainly focused on discrepancies in mothers’ versus adolescents’ ratings of family functioning [3,13,33,34]. However, female patients with a mental illness were still overrepresented in our sample, and future studies should consider a more balanced gender ratio.

Additional directions for future research regard possible predictors and the underlying mechanisms of discrepancies in families affected by parental mental illness. To investigate the development of discrepancies, longitudinal research is essential [28]. Furthermore, recent studies have shown that discrepancies in family members’ perceptions of family functioning can have a predictive value for child outcomes in physical [28] and psychological health domains [30,65]. Applying this line of research to the context of parents with a mental illness is especially relevant since their children represent a high-risk group for the development of health-related problems [5,6,7,8].

## 5. Conclusions

Our results suggest that family functioning is impaired in families affected by parental mental illness. Family-based interventions should be offered to affected families since they address family issues and have been shown effective in improving family functioning. Mentally ill parents view their family as more dysfunctional than their partners and children on several domains, whereas ratings of partners and children are more similar. Family members’ ratings are moderately associated with external ratings of clinicians. In research and therapy, ratings of family functioning should not only be obtained by mentally ill parents but also their partners or children as well as clinicians since this allows for a more comprehensive understanding of the family.

## Figures and Tables

**Table 1:** Demographic and clinical characteristics of patients, partners, and children.

	Patients (*N* = 207)	Partners (*N* = 139)	Children (*N* = 100)
	*n* (%)	*n* (%)	*n* (%)
Gender (female)	156 (75.4)	51 (36.7)	64 (64.0)
Living with both parents			53 (53.0)
Marital status			
Married	111 (54.4)	101 (72.7)	
Unmarried	53 (26.0)	22 (15.8)	
Divorced/Widowed	40 (19.7)	16 (11.5)	
School leaving certificates			
Higher education entrance qualification	63 (31.7)	52 (38.8)	
Intermediate school certificate	89 (44.7)	51 (38.1)	
Compulsory basic secondary schooling	41 (20.6)	29 (21.6)	
No school leaving certificate	4 (2.0)	1 (0.7)	
Psychiatric disorders (ICD-10) ^1^			
F10–F19	3 (1.4)		
F20–F29	10 (4.8)		
F30–F39	119 (57.5)		
F40–F48	25 (12.1)		
F60–F69	49 (23.7)		
F90–F98	1 (0.5)		
Comorbid psychiatric disorders ^1^	84 (40.6)		
Lifetime psychiatric hospitalization	152 (74.5)	18 (13.0)	
Current psychotherapeutic treatment	137 (67.5)	18 (13.0)	9 (9.4)

Note. ^1^ Rated by attending clinician; F10–F19 = Mental and behavioral disorders due to the use of psychoactive substances; F20–F29 = Schizophrenia, schizotypal and delusional disorders; F30–F39 = Affective disorders; F40–F48 = Neurotic, stress-related and somatoform disorders; F60–F69 = Disorders of personality and behavior in adult persons; F90–F98 = Behavioral and emotional disorders with onset usually occurring in childhood and adolescence.

**Table 2:** Descriptive statistics of the FAM for the different family members.

		Patients (*N* = 207)	Partners (*N* = 139)	Children (*N* = 100)
		***M (SD)***	***M (SD)***	***M (SD)***
Raw scores	Global Family Functioning	36.86 (15.34)	30.98 (13.34)	33.22 (14.57)
	Task Accomplishment	6.13 (2.72)	5.00 (2.45)	5.32 (2.74)
	Role Performance	6.78 (2.71)	5.77 (2.64)	6.02 (2.40)
	Communication	5.08 (2.72)	3.99 (2.26)	4.66 (2.68)
	Emotionality	4.82 (2.35)	4.22 (2.17)	4.67 (2.61)
	Affective Involvement	4.66 (2.90)	4.01 (2.54)	4.03 (2.66)
	Control	4.91 (2.72)	4.18 (2.58)	4.32 (2.31)
	Values and Norms	4.49 (2.51)	3.81 (2.27)	4.20 (2.50)
T-scores	Global Family Functioning	66.30 (14.96)	59.58 (12.00)	54.44 (12.13)
	Task Accomplishment	63.07 (11.96)	58.73 (11.21)	56.80 (11.70)
	Role Performance	57.95 (9.57)	54.99 (9.14)	51.03 (8.68)
	Communication	68.86 (17.36)	59.24 (12.63)	55.51 (12.69)
	Emotionality	62.94 (12.91)	59.10 (11.94)	55.24 (11.89)
	Affective Involvement	63.13 (15.97)	60.19 (14.13)	53.84 (12.12)
	Control	59.86 (14.66)	55.47 (12.96)	50.01 (10.22)
	Values and Norms	59.15 (13.90)	54.67 (12.05)	51.65 (11.65)

Note. Based on raw scores and T-scores; FAM = Family Assessment Measure.

**Table 3:** Overall family members’ ratings of family functioning (FAM) compared to normative data.

	Model-Based		
	Adjusted Mean	95% CI	*p*
Global Family Functioning	62.04	[60.42, 63.67]	<0.001
Task Accomplishment	60.37	[59.03, 61.71]	<0.001
Role Performance	55.69	[54.64, 56.74]	<0.001
Communication	63.56	[61.77, 65.34]	<0.001
Emotionality	60.12	[58.70, 61.55]	<0.001
Affective Involvement	60.53	[58.79, 62.26]	<0.001
Control	56.80	[55.27, 58.35]	<0.001
Values and Norms	56.44	[54.95, 57.93]	<0.001

Note. *N* = 210 families with ratings of 207 patients, 139 partners, and 100 children; calculations were based on average T-scores and analyzed with linear mixed models; FAM = Family Assessment Measure; CI = confidence interval.

**Table 4:** Differences between family members’ ratings of family functioning.

	Overall Fixed Effect of Family Member	Comparison between Patient and Partner			Comparison between Patient and Child			Comparison between Partner and Child		
	*p*	Mean Difference	95% CI	*p*	Mean Difference	95% CI	*p*	Mean Difference	95% CI	*p*
Global Family Functioning	<0.001	5.11	$\left[2.75,\;7.47\right]$	<0.001	5.90	$\left[3.11,\;8.70\right]$	<0.001	0.79	$\left[-2.20,\;3.78\right]$	0.603
Task Accomplishment	<0.001	1.02	$\left[0.58,\;1.47\right]$	<0.001	1.14	$\left[0.61,\;1.67\right]$	<0.001	0.12	$\left[-0.45,\;0.68\right]$	0.684
Role Performance	<0.001	0.89	$\left[0.43,\;1.35\right]$	<0.001	1.16	$\left[0.61,\;1.70\right]$	<0.001	0.27	$\left[-0.31,\;0.85\right]$	0.364
Communication	<0.001	1.05	$\left[0.59,\;1.51\right]$	<0.001	0.77	$\left[0.23,\;1.31\right]$	0.005	−0.28	$\left[-0.86,\;0.29\right]$	0.337
Emotionality	0.014	0.60	$\left[0.19,\;1.01\right]$	0.004	0.35	$\left[-0.13,\;0.83\right]$	0.151	−0.25	$\left[-0.77,\;0.26\right]$	0.338
Affective Involvement	0.002	0.56	$\left[0.10,\;1.02\right]$	0.018	0.90	$\left[0.36,\;1.44\right]$	0.001	0.34	$\left[-0.24,\;0.92\right]$	0.252
Control	0.002	0.58	$\left[0.11,\;1.05\right]$	0.016	0.91	$\left[0.36,\;1.46\right]$	0.001	0.33	$\left[-0.26,\;0.91\right]$	0.277
Values and Norms	0.039	0.55	$\left[0.12,\;0.98\right]$	0.013	0.35	$\left[-0.15,\;0.86\right]$	0.171	−0.20	$\left[-0.74,\;0.35\right]$	0.478

Note. *N* = 210 families with ratings of 207 patients, 139 partners, and 100 children; calculations were based on raw scores and analyzed with linear mixed models; pairwise comparisons are based on estimated marginal means; CI = confidence interval.

## Data Availability

The data presented in this study are available on request from the corresponding authors.
